# Dynamic Interactive Social Cognition Training in Virtual Reality (DiSCoVR) for People With a Psychotic Disorder: Single-Group Feasibility and Acceptability Study

**DOI:** 10.2196/17808

**Published:** 2020-08-07

**Authors:** Saskia Anne Nijman, Wim Veling, Kirstin Greaves-Lord, Maarten Vos, Catharina Elizabeth Regina Zandee, Marije Aan het Rot, Chris Neeltje Wil Geraets, Gerdina Hendrika Maria Pijnenborg

**Affiliations:** 1 Department of Psychotic Disorders GGZ Drenthe Assen Netherlands; 2 University Center of Psychiatry University Medical Center Groningen University of Groningen Groningen Netherlands; 3 Department of Clinical and Developmental Neuropsychology Faculty of Behavioral and Social Sciences University of Groningen Groningen Netherlands; 4 Department of Child & Adolescent Psychiatry/Psychology Erasmus MC-Sophia Rotterdam Netherlands; 5 Autism Team Northern-Netherlands of Jonx Department of (Youth) Mental Health and Autism Lentis Psychiatric Institute Groningen Netherlands; 6 Department of Yulius Autism Yulius Dordrecht Netherlands; 7 Department of Clinical Psychology and Experimental Psychopathology Faculty of Behavioral and Social Sciences University of Groningen Groningen Netherlands; 8 Flexible Assertive Community Treatment Team Outpatient treatment center GGZ Delfland Delft Netherlands

**Keywords:** social cognition, virtual reality, psychotic disorder, cognitive remediation therapy, emotion perception, theory of mind, social cognition training

## Abstract

**Background:**

People with a psychotic disorder commonly experience problems in social cognition and functioning. Social cognition training (SCT) improves social cognition, but may inadequately simulate real-life social interactions. Virtual reality (VR) provides a realistic, interactive, customizable, and controllable training environment, which could facilitate the application of skills in daily life.

**Objective:**

We developed a 16-session immersive VR SCT (Dynamic Interactive Social Cognition Training in Virtual Reality [DiSCoVR]) and conducted a single-group feasibility pilot study.

**Methods:**

A total of 22 people with a psychotic disorder and reported problems in social cognition participated. Feasibility and acceptability were assessed using a survey for participants and therapists, and by examining relevant parameters (eg, dropouts). We analyzed preliminary treatment effects on social cognition, neurocognition, and psychiatric symptoms.

**Results:**

A total of 17 participants completed the study. Participants enjoyed DiSCoVR (mean 7.25, SD 2.05; range 3-10), thought it was useful for daily social activities (mean 7.00, SD 2.05; range 3-10), and enjoyed the combination of VR and a therapist (mean 7.85, SD 2.11; range 3-10). The most frequently mentioned strength of DiSCoVR was the opportunity to practice with personalized social situations (14/20, 70%). A significant improvement of emotion perception was observed (Ekman 60 Faces; t_16_=–4.79, *P*<.001, *d*=–0.67), but no significant change was found in other measures of social cognition, neurocognition, psychiatric symptoms, or self-esteem.

**Conclusions:**

DiSCoVR was feasible and acceptable to participants and therapists, and may improve emotion perception.

## Introduction

People with a psychotic disorder commonly experience problems with social functioning, that is, impairments in the ability to interact successfully with the social environment and to adequately fulfil a societal role (eg, work, personal relationships) [1]. These are often related to problems in the cognitive processes used in understanding and thinking about interactions with other people, known as social cognition [2,3].

The most commonly identified domains of social cognition are emotion perception (ie, the identification and processing of emotional cues), social perception and social knowledge (understanding social cues, rules, and context), theory of mind (ToM; identifying and understanding others’ mental states and separating these from one’s own perspective), and attribution style (inferences about the causes and intentions underlying events and others’ behavior) [4]. A meta-analysis found moderate to large deficits in people with schizophrenia in emotion perception, social perception, and ToM, but not in attribution style [2].

Social cognition has become an important treatment target for improvement of social functioning; a multitude of behavioral approaches to improve social cognition and social functioning has emerged in recent years [5]. Three meta-analyses have found moderate to large effects of social cognition training (SCT) interventions on social cognition [6-8]. Broad-based or comprehensive forms of SCT (eg, Social Cognition and Interaction Training [9]) appear to be the most effective overall [8]. Improvements in social functioning were found in 2 meta-analyses [6,8], though only for broad-based SCT in the latter meta-analysis [8].

However, there are some concerns about SCT [5]. First, while positive effects on lower-order social cognitive domains are robust, findings regarding higher-order domains are more heterogeneous, and depend on the type of task used [5]. Second, findings regarding the generalization of social cognitive gains, as measured by social cognition tasks, to daily life social functioning are mixed; many studies do not find a significant effect [5]. Third, the durability of treatment effects has not yet been established; of the available studies, some find sustained improvements at follow-up, whereas others do not [5,8]. Thus, while SCT has a demonstrated effect on social cognition, particularly lower-order domains, these improvements do not always enduringly carry over to higher-order social cognitive domains and social functioning.

One possible explanation for these mixed findings could be that the generalization of training material to social functioning requires better opportunities to apply training techniques in real-life social situations. From studies on cognitive remediation, we know that combining treatment with a meaningful context to practice newly learned behavior is vital for improvement of social functioning [10]. However, the techniques and stimuli that are typically used in SCT, such as group discussions, videos, and pictures, lack the complexity and dynamic interaction that are present in real-life social situations [5,11]. Although it is theoretically possible to accompany patients and practice in real-life social situations, doing so is generally not feasible in clinical practice. Furthermore, real-life situations cannot be controlled for training purposes.

These shortcomings of SCT could be addressed by administering interventions using virtual reality (VR). VR involves wearing a headset that projects continuously rendered 3D images [12]. With VR, highly immersive, dynamic, and interactive social environments can be created, providing a high degree of ecological validity for assessment as well as treatment [13]. Furthermore, VR is controllable, facilitating structured SCT in realistic social situations, and allows for scenarios to be personalized, repeated, and varied. Therapists can observe participants unobtrusively and provide real-time feedback. In addition, VR has practical benefits, because a wide scope of social situations can be simulated without leaving the treatment setting. Finally, barriers to practice may be smaller with VR than in real life, as users know that their actions have no real-world consequences, and that the VR can be stopped at any time.

VR has been found to be an effective tool in treatment of psychotic disorders, for example, for treating paranoid ideation [14] and auditory verbal hallucinations [15]. A recent pilot study (n=19) of SCT using virtual environments in people with first-episode psychosis reported that SCT in a virtual world was acceptable and feasible, and found improvements in emotion recognition and anxiety. However, no significant change was observed in other domains of social cognition and social functioning [16]. Besides, a case series (n=2) of VR SCT in people with psychotic disorder reported improvements in social cognition and social functioning [17].

Furthermore, 2 trials studied the effect of VR social skills training (SST) on participants with a psychotic disorder: Park and colleagues [18] demonstrated enhanced improvement of assertiveness and conversational skills of a VR SST compared with conventional SST, and a pilot study [19] showed improvements in social anxiety, social functioning, and emotion perception after VR SST. Finally, promising results of VR SCT have been reported in other clinical populations, particularly in those with autism spectrum disorder [20,21]. Together, these encouraging preliminary findings support the viability and utility of VR SCT.

We have developed a VR SCT called “Dynamic Interactive Social Cognition Training in Virtual Reality” (DiSCoVR). In this pilot study, our aims were twofold:

- To determine whether providing VR SCT is feasible and acceptable to participants and therapists, evaluated in terms of commonly used criteria for feasibility (ie, acceptability, user satisfaction, demand, perceived usefulness, implementation potential, practicality, and perceived ease of use [22-24]).
- To explore the effect of DiSCoVR on social cognition, neurocognition, and psychiatric symptoms, by examining participants’ baseline and posttreatment scores.

## Methods

### Design and Participants

This pilot study had a pretest posttest design with a single treatment group. All participants continued to receive their treatment as usual alongside their participation in the study. People with a psychotic disorder were recruited from 3 mental health treatment centers in the Netherlands (University Medical Center Groningen, GGZ Drenthe, and GGZ Delfland). Potential participants were referred to the study by their treating clinician. To help clinicians determine which patients might be eligible, screening questions were provided: (1) *Does this person struggle to recognize what goes on in another person’s mind?,* (2) *Are there observable deficits in their assessment of social situations?,* (3) *Does this person have problems understanding what other people mean?*, and (4) *Do these problems lead to social dysfunction?* Promotional flyers and posters were also distributed. Participants received a compensation of €15 (US $17) for each completed assessment (up to €30 [US $34] if they completed the study), and reimbursement of any travel costs incurred for the assessments.

Inclusion criteria were (1) a diagnosis of a psychotic disorder as determined by a structured diagnostic instrument (eg, Mini-International Neuropsychiatric Interview [M.I.N.I.] [25]) in the past 3 years, or as verified by a structured clinical interview (M.I.N.I. Plus) at baseline; (2) problems in social cognition as indicated by the treating clinician; and (3) an age between 18 and 65 years. Exclusion criteria were (1) an estimated IQ below 70; (2) substance dependence; (3) a diagnosis of a neurological disorder, such as epilepsy or dementia; and (4) inadequate Dutch language proficiency.

### Intervention

DiSCoVR consisted of sixteen 45-60-minute individual treatment sessions, which took place two times a week. The intervention was provided on-site by therapists with (at minimum) a clinical psychology master’s degree. A treatment protocol was used; all therapists were trained in its use. The protocol included background information, examples of goals and strategies, software manuals, exercises (eg, standard situations to practice in role play and their relation to social cognition), and detailed instructions on how to carry out sessions. Therapists received supervision at least once for each client and could consult the research team as needed for additional supervision and technical support.

**Figure 1:**
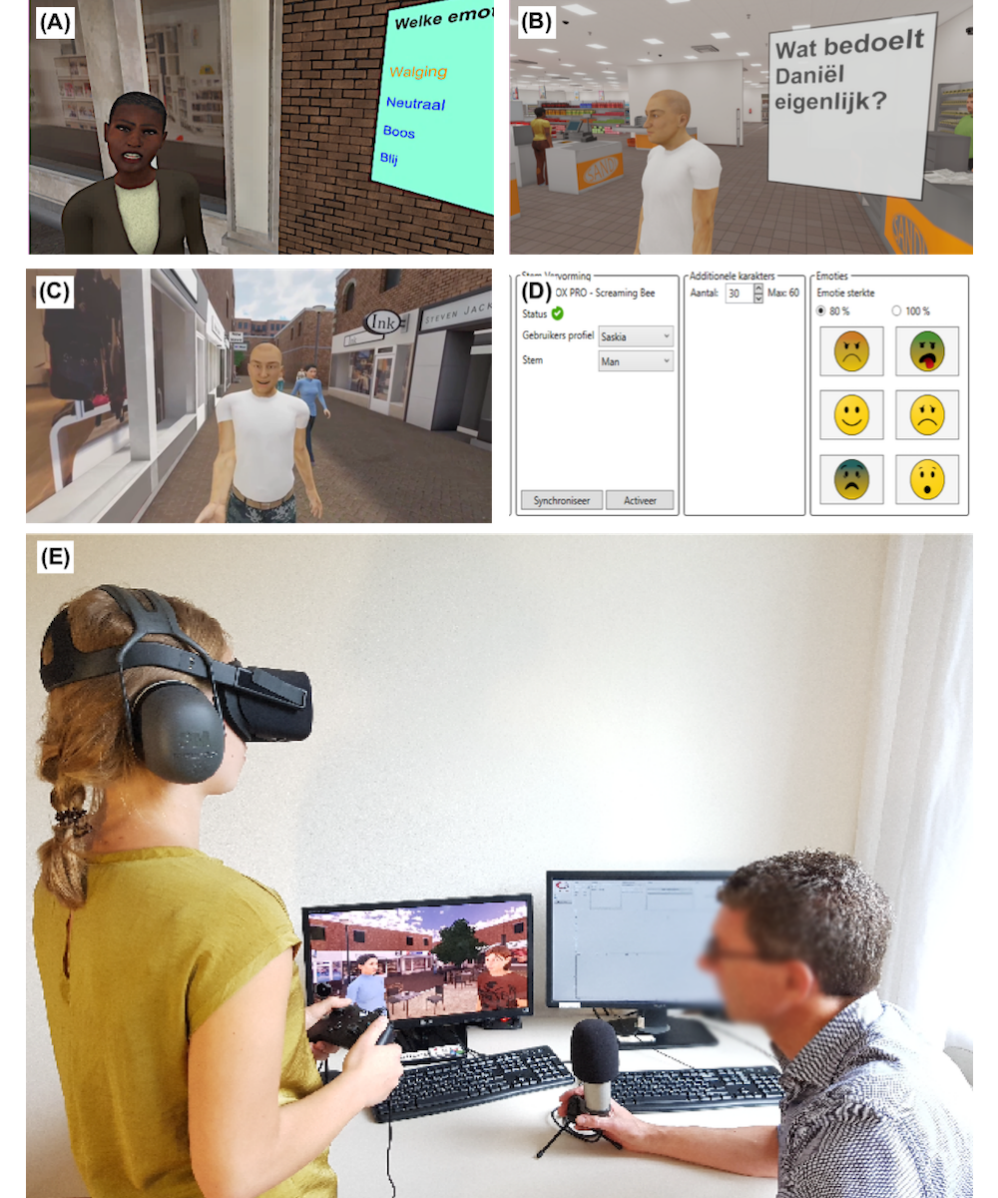
(A) Module 1 (Translation: "Which emotion? Disgust, Neutral, Anger, Happiness"). (B) Module 2 (Translation: "What does Daniel really mean?"). (C) Module 3, participants’ point of view, (D) Module 3, therapist interface. (E) Set-up with authors WV and CNWG, who both consented to publication of this image. Images A-D are used with permission from CleVR BV.

Social cognition was trained by practicing with social material in immersive virtual environments (Figure 1) and by learning to apply strategies in these environments (eg, verbalizing facial characteristics, or verifying with others whether a social assessment is correct). Participants formulated concrete personal goals that could be achieved with improvement of social cognition. At the end of each session, participants reflected on how they could use new knowledge and skills to achieve their goals. In (optional, although strongly encouraged) homework assignments throughout the intervention, participants were encouraged to apply strategies in daily life. The intervention was structured to start with lower-order social cognition, and complexity was increased in each module.

Module 1 (Sessions 1-5) targeted emotion perception, practiced using a VR facial emotion recognition task. Participants explored a shopping street with avatars (virtual characters) who showed an emotion upon approach. Participants then selected the emotion they thought the avatar expressed in a multiple-choice menu. Immediate visual feedback on the correctness of their answer (green or red screen) was given. If an answer was incorrect, the same emotion was shown again with greater intensity. Several characteristics of the avatars and environment (eg, intensity of emotions, allotted time for answers) could be altered. Seven standard practice levels were created in which all available parameters increased in difficulty, but therapists could customize these parameters to create tailored levels. Participants learned strategies to recognize emotions (eg, verbalizing salient features) and practiced them both in VR and in their (real-life) home environment.

Module 2 (Sessions 6-9) targeted social perception and ToM. By practicing with interactive social scenarios, participants learned to understand the social context, hints, social missteps and ambiguity, perception of body language, and tone of voice. Participants observed social interactions between avatars in a café and supermarket, containing misunderstandings, hints, true and false beliefs, and social missteps. They answered multiple-choice and open-ended questions within the VR environment about the emotions, thoughts, and intentions of the avatars. Outside of the VR environment, participants continued to practice strategies (eg, remembering your thoughts/emotions in a similar situation) and tried to assess behavior, thoughts, and emotions of themselves and others in (real-life) daily social situations.

Module 3 (Sessions 10-16) targeted application of higher-order social cognition in social interactions, practiced using interactive role-play exercises. Participants interacted with an avatar, whose appearance, voice, and emotions were controlled by the therapist. Participants practiced with situations that were difficult for them or that fit their goals. Therapists could also use standard (nonpersonalized) role-play exercises from the protocol, containing sarcasm, hinting, misunderstandings, and social missteps. Participants learned a social cognitive problem-solving technique in which they first considered the behavior, thoughts, and emotions of themselves and the other person, then formulated (and role-played) different possible reactions, and finally executed the reaction they preferred. Participants were encouraged to also apply this technique in their daily lives.

The virtual environments (a shopping street, a supermarket, and a bar) were shown using an Oculus Rift VR-headset (Consumer Version 1). The software was developed by CleVR BV. The VR software was controlled by the therapist, using one monitor to observe the participant’s field of vision, and another monitor to control the virtual environment with the user interface. Participants used a Microsoft Xbox game controller to move around and to indicate answers in multiple-choice-menus.

### Measures

#### Diagnostic Measures

The following measures were administered at baseline for diagnostic purposes.

##### Mini International Neuropsychiatric Interview Plus

A semistructured interview—M.I.N.I. Plus [25]—was used to verify the diagnosis of psychotic disorder, if a diagnosis of psychosis had not been determined by a (semi)structured clinical interview (eg, Structured Clinical Interview for DSM, Schedules for Clinical Assessment in Neuropsychiatry, M.I.N.I.) in the past 3 years. This was the case for 17 of our 22 participants (77%).

##### National Adult Reading Test

National Adult Reading Test [26] (Dutch version, Nederlandse Leestest voor Volwassenen, [27]) is a proxy measure of premorbid intelligence. Participants recite a list of 50 increasingly uncommon words. Correct pronunciations yield 2 points.

#### Feasibility and Acceptability

Feasibility and acceptability of DiSCoVR were assessed in participants by a questionnaire consisting of 2 parts: statements about the intervention (eg, “I enjoyed the training”) that were rated on 10-point Likert scales, and open-ended interview questions (eg, “What were strengths of the intervention?”). The complete questionnaire can be found in Tables 2 and 3. We also recorded dropout rate and number of sessions completed as well as the time taken to complete DiSCoVR.

In addition, therapists completed an open-ended questionnaire about their satisfaction with the treatment protocol and materials (Table 4). Protocol fidelity was assessed with a self-report form and checklist after each treatment session, on which therapists could indicate any particularities, whether they deviated from the protocol, and why.

#### Social Cognition

##### Ekman 60 Faces Test

Ekman 60 Faces Test [28] is a 60-item computerized picture task, measuring emotion perception. Participants are asked to identify 6 basic emotions (happy, surprised, anxious, disgusted, sad, and angry). The total score (ie, the number of correctly identified stimuli) across all emotions was analyzed.

##### Bell–Lysaker Emotion Recognition Test

Bell–Lysaker Emotion Recognition Test (BLERT [29], Dutch version unpublished) is a video task measuring emotion perception, consisting of 35 sentences, in which actors portray an emotionally ambiguous sentence neutrally or with 1 of 6 basic emotions. Participants identify the portrayed emotion. Total scores were analyzed.

##### The Awareness of Social Inference Task

The Awareness of Social Inference Task (TASIT [30]; Dutch version [31]) is a video task containing social vignettes, and consists of 3 parts: I-III. TASIT-I assesses emotion perception, distinguishing between neutral and 6 basic emotions. TASIT-II and -III measure social perception and ToM, and have questions about the intentions, message, thoughts, and feelings of the people in the video. TASIT-II consists of clips with genuine utterings or sarcasm; TASIT-III contains clips of lies or sarcasm. Parallel versions were used; the version order was A-B for all participants. For analysis, we used the total score for each part of TASIT.

##### Empathic Accuracy Task

Empathic Accuracy Task (EAT [32]; Dutch version [33]) is a computerized video task measuring empathy, with clips of people speaking about emotionally charged autobiographical events with either a positive or a negative valence. Conforming to previous studies [34,35], a shortened version (4 videos; 2 positive and 2 negative) was used. Parallel versions were administered, using counterbalanced randomization. Participants used a rating dial to indicate continuously how speakers were feeling while speaking (very negative to very positive). Empathic accuracy scores were generated for each video clip by correlating participants’ affect ratings with original speakers’ own affect ratings. These correlations (–1 to +1) underwent a Fisher *z* transformation prior to data analysis. For each participant, the mean Fisher *z* transformed EAT scores across video clips were used in the data analyses.

##### Faux Pas

Faux Pas [36] is a measure of ToM. Ten stories are read to the participant, 5 of which contain a faux pas. Participants are asked whether a faux pas occurred, who committed it, why it was a faux pas, and why it happened. A story comprehension and empathy question are also asked after each story. Parallel versions were used, but the order was not counterbalanced or randomized. The total score was used for analysis.

#### Neurocognition

##### Rapid Visual Processing

Rapid Visual Processing (RVP; [37]) is a measure of sustained visual attention. A white box with alternating numbers (0-9, 100 digits per minute) is shown on a computer screen. Participants press a button if 1 of the 3 target sequences occur. Outcome variables of the RVP are response latency in milliseconds, sensitivity, and probability of hit (0-1).

##### Trail Making Test

The Trail Making Test (TMT; [38]) assesses processing speed and executive function. Numbers (TMT-A) or numbers and letters (TMT-B) are shown in circles, scattered across a sheet of paper. Participants connect the numbers (and letters) in consecutive order (eg, 1-2-3 or 1-A-2-B). The completion time in seconds for each subtest was used for analysis.

#### Symptom Measures

##### Green Paranoid Thought Scale

The Green Paranoid Thought Scale (GPTS; [39]) is a 32-item self-report questionnaire measuring paranoid thoughts on 2 dimensions (social reference and social persecution), using a 5-point Likert scale. We analyzed the total score for both subscales separately.

##### Social Interaction Anxiety Scale

The Social Interaction Anxiety Scale (SIAS; [40]) is a 20-item self-report questionnaire investigating verbal and nonverbal social anxiety, using a Likert scale: 0 (not at all) to 4 (completely). The total score was analyzed.

##### Beck Depression Inventory

Beck Depression Inventory (BDI; [41]) is a 21-item self-report questionnaire on symptoms of depression. Each item of the BDI uses statements fitting an increasing severity of depressive symptoms. We used the total BDI score for analysis.

##### Self-Esteem Rating Scale

The Self-Esteem Rating Scale (SERS; [42]) is a 20-item self-report questionnaire on (explicit) self-esteem. The SERS uses statements that are rated on a 1 (disagree totally) to 7 (agree totally) Likert scale. The total score was used for analysis.

##### Positive and Negative Syndrome Scale

The Positive and Negative Syndrome Scale (PANSS; [43]) is a semistructured interview investigating symptoms of psychosis. The positive (7 items) and negative (7 items) subscales were administered. Total subscale scores were used for analysis.

### Procedure

After referral from a clinician or self-enrollment, interested patients were contacted and screened by the research team. Participants provided written informed consent during a face-to-face meeting, after which the baseline assessment (approximately 3 hours) took place. An overview of the measures, including their order and length, is included in Multimedia Appendix 1. Measurements were performed by trained assessors. After the baseline measurement, participants were enrolled in DiSCoVR. Upon finishing the training, a (face-to-face) posttreatment assessment (approximately 2.5 hours) took place. Participants who dropped out were asked to participate in the evaluation survey.

This study was approved by the Medical Ethical Committee of the University Medical Center Groningen (ABR: NL55477.042.16, METC: 2016/050), as well as by the ethics boards of the other participating centers (ie, the Committee for Research and Health Care Innovation, GGZ Drenthe, the Committee for Scientific Research, and GGZ Delfland). All participants gave written informed consent in accordance with the Declaration of Helsinki.

### Analysis

We assessed 3 types of feasibility and acceptability data: (1) relevant quantitative parameters, such as dropout rates, time to recruit, intervention completion time, protocol adherence, and occurrence of issues (eg, technical problems) in sessions; (2) the participant survey; and (3) the therapist survey.

For quantitative data, descriptive statistics were examined. For qualitative items, similar answers or categories were grouped together and absolute and relative frequencies were evaluated. That is, because questions were open ended, we grouped comparable answers and counted how frequently they occurred. For example, for the question “Did the training meet your treatment needs?”, the answers “I’ve learned what I’d wanted to learn” and “After setting goals, I could work on them very well” were grouped as “Yes,” whereas “I did not fully succeed in developing better empathy, but I was able to practice,” and “On some points, but not others” were grouped as “Partly”.

To compare baseline and posttreatment scores, paired *t* tests were used, unless difference scores (T_1_–T_0_) were not normally distributed. This was the case for the BLERT, GPTS-A, PANSS Positive, and TMT-B. Thus, Wilcoxon tests were carried out for these measures. Pairwise complete-case analysis was used in case of missing data. To account for the multitude of measures, we adopted an α of .01 as a threshold of significance.

## Results

### Feasibility and Acceptability

#### Participants

Demographic and clinical characteristics of the sample are presented in Table 1. Participants were recruited between January and August 2017; 17 of the 22 participants (77%) completed the study. Reasons for dropout (n=5) were having too much going on (n=2), finding the intensity too high (n=1), not feeling a connection with the therapist (n=1), and (self-reported) negative symptoms and social anxiety (n=1). Noncompleters dropped out at Sessions 2 (n=1), 4 (n=1), 7 (n=2), and 10 (n=1). Three of the five participants who dropped out participated in the evaluation survey.

**Table 1:** Demographic and clinical characteristics of the sample (N=22).

Variables			Value
**Demographic**			
	Age (years)		35.95 (11.68)
	**Gender**		
		Male, n (%)	16 (73)
		Female, n (%)	6 (27)
	**Education level**		
		None or primary, n (%)	1 (5)
		Vocational, n (%)	12 (55)
		Secondary, n (%)	8 (36)
		Higher, n (%)	1 (5)
	National Adult Reading Test score, mean (SD)		78.64 (7.70)
	**Paid employment**		
		Employed, n (%)	4 (18)
		Unemployed, n (%)	18 (82)
		Hours worked per week, mean (SD)	1.14 (3.12)
		Work history (years), mean (SD)	6.52 (9.91)
	**Day activities/volunteering**		
		Engages in day activities/volunteering, n (%)	12 (55)
		Hours spent on day activities/volunteering, mean (SD)	5.7 (8.02)
	**Substance use**		
		Alcohol (glasses per week), mean (SD)	3.55 (5.55)
		Nicotine (cigarettes per week), mean (SD)	63.45 (90.12)
		Marijuana/Cannabis (joints per week), mean (SD)	0.50 (1.67)
		Hard drugs (eg, ecstasy, speed, cocaine) in standard units per week, mean (SD)	0.10 (0.44)
**Clinical**			
	**Diagnosis**		
		Schizophrenia, n (%)	17 (77)
		Schizoaffective disorder, n (%)	2 (9)
		Psychotic disorder, not otherwise specified, n (%)	3 (14)
	**Hospitalization status**		
		Never hospitalized, n (%)	3 (14)
		Currently hospitalized, n (%)	3 (14)
		Previously hospitalized, n (%)	16 (73)
	Number of (past) psychotic episodes, mean (SD)		2.31 (1.8)
	Illness duration, mean (SD)		13.22 (11.70)
	**Medication**		
		Typical antipsychotics, n (%)	0 (0)
		Atypical antipsychotics, n (%)	13 (59)
		Combination typical/atypical, n (%)	1 (5)
		Antidepressants, n (%)	1 (5)
		Mood stabilizers, n (%)	1 (5)
		Benzodiazepines, n (%)	1 (5)
		Not using medication, n (%)	2 (9)
		Unknown/declined to answer, n (%)	5 (23)
	**Family history of psychiatric illness**		
		Yes, n (%)	12 (55)
		No, n (%)	10 (45)

The results of the survey are presented in Tables 2 and 3. In the quantitative survey, participants gave positive ratings to their enjoyment of DiSCoVR (mean 7.25, SD 2.05; range 3-10), to the amount they learned (mean 6.65, SD 1.81; range 3-10), and usefulness for daily social activities (mean 7.00, SD 2.05; range 3-10). Participants positively evaluated the combination of a therapist and VR (mean 7.85, SD 2.11; range 3-10) and the appropriateness of the difficulty level (mean 7.20, SD 1.91; range 3-10). Participants gave relatively low ratings to the realism of the appearance of the avatars (mean 5.45, SD 2.18; range 2-10) but ratings for their facial expressions (mean 6.65, SD 2.06; range 3-10) and voices (mean 6.95, SD 2.35; range 3-10) were higher.

In the open-ended questions of the qualitative survey (N=20), participants most commonly (n=14, 70%) mentioned the opportunity to practice with social situations in VR as a strength of the intervention. Other common subjective strengths of DiSCoVR were the personalization of the intervention (ie, targeting specific personal goals and situations; n=5, 25%) and (role of) the therapist (n=5, 25%), the emotion recognition module (n=3, 15%), and realism of emotions and role-play exercises (n=3, 15%). For a majority of people (n=13, 65%), the intervention fit their treatment needs. The most commonly reported subjective effect of DiSCoVR was improved social skills (n=7, 35%), followed by improved emotion recognition (n=6, 30%) and increased assertiveness and confidence (n=5, 25%).

The aspect of DiSCoVR most commonly named as a weakness was technical issues (n=7, 35%), particularly problems with sound regulation in the interaction module, as well as limitations of the content (eg, the inability to practice group conversations) and the graphical quality. Some participants criticized the realism of the intervention (n=4, 20%), particularly the avatars’ movements and facial expressions. A few participants (n=4, 20%) indicated that the treatment only partly fit their needs; reasons given were that they felt their social cognition had improved, but not as much as they had wanted (n=1); that the latter part of the training was useful, but not the emotion perception module (n=1); that it was relevant and they had learned useful strategies, but that the intervention could be more focused and that they needed to keep reminding themselves to use them (n=1); and that there was insufficient opportunity to practice “small talk” (n=1). Overall, 3/20 (15%) participants stated that the intervention did not fit their needs: one participant felt it was too focused on (others’) behavior; one indicated that recognizing emotions in conversations was still difficult; and one thought the role-play exercises were insufficiently realistic.

Finally, while a majority indicated that they were satisfied with the number (n=10, 50%), intensity (n=5, 25%), and duration (n=13, 65%) of sessions, those who were employed or had a long commute found the intensity too high (n=1, 5%) or somewhat high (n=4, 20%).

**Table 2:** Quantitative evaluation of VR intervention by participants (N=20).

Question	Mean (SD)	Median	Range	IQR (Q1-Q3)
I liked the VR training (1-10)	7.25 (2.05)	7	3-10	3 (6-9)
I have learned a lot from the VR training (1-10)	6.65 (1.81)	7	3-10	2 (6-8)
I thought the training was useful for daily social contact (1-10)	7.00 (2.05)	7.5	3-10	1.75 (6.25-8)
I thought the virtual characters looked realistic (1-10)	5.45 (2.18)	5.5	2-10	3 (4-7)
I thought the virtual characters’ voices sounded realistic (1-10)	6.95 (2.35)	7	2-10	3.5 (5.25-8.75)
I thought the virtual characters’ facial expressions looked realistic (1-10)	6.65 (2.06)	6.5	3-10	3.75 (5-8.75)
I enjoyed the combination of a therapist and VR (1-10)	7.85 (2.11)	8.5	3-10	3.5 (6.25-9.75)
The difficulty level of the training was exactly right (1-10)	7.20 (1.91)	7.5	3-10	2.75 (6-8.75)

**Table 3:** Qualitative evaluation of the VR intervention by participants (N=20).

Question and answers			n (%)^a^	
**Were you satisfied with the number, intensity, and duration of sessions?**				
	**Number**			
		Satisfied		10 (50)
		Too many		3 (15)
		Too few		1 (5)
	**Intensity**			
		Acceptable		5 (25)
		Somewhat high		4 (20)
		Too high		1 (5)
	**Duration**			
		Satisfied		13 (65)
		Too long		3 (15)
		Too short		1 (5)
**What were strengths of the intervention?**				
	Practice with social situations/interaction module		14 (70)	
	Tailoring of intervention to personal situation		5 (25)	
	(Role of) therapist		5 (25)	
	Emotion recognition module		3 (15)	
	Realism		3 (15)	
	Techniques and materials		3 (15)	
	Other (ie, structure, second module, assessment)		3 (15)	
**What were weaknesses of the intervention?**				
	Technical/sound issues		7 (35)	
	Realism (appearance, movement)		4 (20)	
	First module (too long/unnecessary)		3 (15)	
	Techniques/materials		3 (15)	
	Other (ie, dizziness, assessment duration, therapist, cognitive load, tailoring, too much emphasis on others, difficulty of homework)		6 (30)	
	None		4 (20)	
**What have you learned?**				
	Social skills (talking to others, how to react)		7 (35)	
	Recognize emotions		6 (30)	
	Being assertive/confident		5 (25)	
	Paying attention/showing interest		4 (20)	
	Think positively/consider alternative explanations		3 (15)	
	Knowing what is appropriate/being less abrasive		2 (10)	
	Other (ie, revisiting things, less anxiety)		3 (15)	
	Nothing		1 (5)	
**Did the intervention meet your treatment needs?**				
	Yes		13 (65)	
	No		3 (15)	
	Partly		4 (20)	
**What did you think of the conversations of (Module 2) and with (Module 3) the avatars?**				
	Good/realistic/opportunity for learning		11 (55)	
	Okay, needs some improvement		6 (30)	
	Fake/unrealistic		1 (5)	
	Funny/takes getting used to		3 (15)	
	Not applicable		1 (5)	

^a^n (%) refers to the number and percentage of participants who provided a certain answer. Because participants could provide multiple answers to a single question (eg, “I learned social skills and assertiveness”), and some participants did not answer all questions completely (eg, “The number of sessions was fine,” but said nothing about the intensity/duration), n (%) may not add to 20 (100).

#### Therapists

The results of the evaluation of DiSCoVR by therapists are presented in Table 4. Therapists noted the role-play exercises and the opportunity to practice with social situations in VR as the main strength of DiSCoVR (4/6, 67%), considering these to be the most important and effective component of the intervention (5/6, 83%), followed by reflection on social situations (3/6, 50%). Other commonly named strengths were the treatment protocol (3/6, 50%) and the structure of the intervention (2/6, 33%). The majority of therapists considered the VR software to be adequate (4/6, 67%) or good (2/6, 33%), stating that it was easy and intuitive to work with (4/6, 67%), and praising its technical support (2/6, 33%).

The therapists mainly criticized the lack of technical reliability and limited capabilities of the software (5/6, 83%). Half of them recommended improving existing functionality, particularly the sound and graphical quality, and half of them recommended adding new features (eg, environments or scenarios). Regarding the relevance of scenarios for daily life, 2/6 (33%) therapists were satisfied, 3/6 (50%) felt they were relevant but could be improved, and 1/6 (17%) was dissatisfied and noted that they felt unnatural.

Therapists reported deviating from the protocol in 18.2% (55/303) of the total number of sessions that were carried out. In addition, technical issues were reported in 14.9% (45/303) of the sessions. Other issues (eg, a participant being late) occurred in 11.6% (35/303) of sessions. A total of 3/303 sessions (0.99%) were terminated early. It took a mean of 12.4 weeks (SD 5.2; range 8-22; median 11) for participants to complete the intervention. The reports indicated that participants spent a mean of 342 minutes practicing in VR across the 16 sessions (SD 28.8; range 227-451; median 362), which is equivalent to a mean of 17.9 minutes per session (SD 6.06; range 5.0-28.2; median 17.9). The mean duration of a session was 55.4 minutes (SD 7.2; range 34-67; median 57).

**Table 4:** Evaluation of the VR intervention by therapists (N=6).

Question and answers		n (%)^a^	
**Was the degree of interaction of the VR SCT adequate?**			
	More than adequate		2 (33)
	Adequate		1 (17)
	Somewhat inadequate		2 (33)
**Are the used scenarios relevant for daily life?**			
	Yes		2 (33)
	Yes, but needs improvement		3 (50)
	No, somewhat unnatural		1 (17)
**Do you consider the difficulty level to be adequate?**			
	Yes		2 (33)
	Adequate		1 (17)
	Needs to be more difficult		1 (17)
	Needs to be less difficult		1 (17)
	Depends on therapist		1 (17)
**What were strengths of the intervention?**			
	Interactive role-play exercises (Module 3)		4 (67)
	Use of VR/Practice with social situations		3 (50)
	(Ease of use of) Protocol		3 (50)
	Structure of intervention		2 (33)
	Techniques/materials		1 (17)
	Enjoyable		1 (17)
**Which components were effective or important?**			
	Role-play exercises (Module 3)		5 (83)
	Evaluation/reflection		3 (50)
	Practicing		1 (17)
	All components		1 (17)
**What were weaknesses or annoyances?**			
	Technical issues/shortcomings		5 (83)
	Lack of reference to participants’ worksheets		2 (33)
	Homework instructions inadequate		1 (17)
	Goal setting difficult		1 (17)
	Module 1 too long		1 (17)
	Scenarios too long		1 (17)
	Thoughts–behavior–feelings technique too difficult		1 (17)
**What did you think of the VR program?**			
	Good		2 (33)
	Adequate, but needs some improvement		4 (67)
	Easy/intuitive interface, easy to work with		4 (67)
	Good technical support		2 (33)
	Not sufficiently advanced graphically		1 (17)
**What did you think of the number and duration of the sessions?**			
	Fine		4 (67)
	Fine, but should be structured differently		1 (17)
	Needs more sessions		1 (17)
	Needs longer sessions		1 (17)

^a^n (%) refers to the number and percentage of therapists who provided a certain answer. Because they could provide multiple answers to a single question and some therapists did not answer all questions completely, n (%) may not add to 6 (100).

### Effects of DiSCoVR (Baseline Versus Posttreatment)

Baseline and posttreatment means, standard deviations, test statistics, and effect sizes are presented in Table 5. Analyses were conducted with 17 participants (unless indicated otherwise). At =.01, only the emotion perception, as measured by the Ekman 60 Faces Test, improved significantly after DiSCoVR (t_16_=–4.79, *P*<.001, mean difference 4.18). No significant improvement was observed on the other measures of emotion perception, any of the ToM measures, in neurocognition, or in levels of psychiatric symptoms.

For emotion perception, a moderate, effect size (*d*=–0.67) was found for the Ekman 60 Faces Test, but for the other measures, effect sizes were negligible (BLERT: *d*=0.03) and small (TASIT-I: *d*=–0.15). For social perception and ToM, we found negligible to small effects on all outcome measures (ranging between *d*=–0.15 and *d*=0.25). Negligible effects were found for information processing (TMT-A and B; *d*=0.11 and *d*=0.08), but small to moderate effects were found for sensitivity and probability of hit of the RVP (*d*=–0.47 for both). Small improvements were also observed for most symptom domains, with effect sizes ranging between *d*=0.16 and *d*=0.34. A small effect size was also found for self-esteem (*d*=–0.25).

**Table 5:** Means, standard deviations, and test statistics (baseline and posttreatment).

Measure		Baseline, mean (SD) N=22	Posttreatment, mean (SD) N=17^a^	*t* or W	*P*	Cohen *d* (95% CI)
**Emotion Perception**						
	Ekman 60 Faces	46.86 (5.95)	51.06 (6.72)	–4.79	<.001^b^	–0.67 (–0.97 to –0.35)
	BLERT^a^	22.18 (5.61)	22.12 (7.97)	38.50^c^	.38	0.03 (–0.41 to 0.48)
	TASIT, Part 1	11.77 (1.77)	12.24 (1.25)	–0.57	.58	–0.15 (–0.71 to 0.40)
**Social perception and Theory of mind**						
	TASIT, Part 2	26.59 (4.52)	26.71 (3.94)	0.55	.59	0.15 (–0.39 to 0.69)
	TASIT, Part 3	22.86 (3.73)	23.76 (4.21)	–0.59	.56	–0.16 (–0.70 to 0.38)
	Faux Pas Test	39.14 (7.62)	37.88 (6.87)	0.89	.39	0.17 (–0.22 to 0.56)
	EAT^a^	1.12 (0.54)	1.24 (0.33)	0.45	.66	0.25 (–0.51 to 0.81)
**Neurocognition**						
	TMT A	39.59 (14.76)	36.41 (16.70)	0.79	.44	0.11 (–0.17 to 0.29)
	TMT B	77.64 (25.13)	78.59 (43.96)	83.5^c^	.42	0.08 (–0.59 to 0.43)
	RVP sensitivity (A′)	0.89 (0.05)	0.91 (0.04)	–2.10	.05	–0.47 (–0.95 to 0.01)
	RVP mean latency	460.54 (121.43)	473.99 (135.37)	0.271	.79	0.03 (–0.26 to 0.34)
	RVP probability of hit	0.56 (0.20)	0.64 (0.17)	–2.21	.04	–0.47 (–0.93 to –0.01)
**Symptoms**						
	Depression (BDI)^a^	14.05 (9.93)	12.50 (7.09)	1.65	.12	0.31 (–0.08 to 0.91)
	Paranoia: social reference (GPTS)	34.09 (14.72)	27.88 (10.40)	84.0^c^	.17	0.34 (–0.18 to 0.87)
	Paranoia: social persecution (GPTS)	29.77 (14.92)	25.82 (15.10)	1.15	.27	0.16 (–0.23 to 0.55)
	Positive symptoms (PANSS)	15.45 (4.27)	13.18 (3.28)	43.5^c^	.72	0.21 (–0.28 to 0.70)
	Negative symptoms (PANSS)	14.55 (3.80)	14.94 (4.59)	0.675	.51	0.17 (–0.34 to 0.67)
	Social anxiety (SIAS)^a^	41.82 (14.74)	34.13 (13.19)	1.59	.13	0.27 (–0.17 to 0.72)
	Self-esteem (SERS)	84.18 (21.08)	90.41 (17.62)	–1.79	.09	–0.25 (–0.67 to 0.17)

^a^Because of missing data, BLERT T_1_, n=16; EAT T_1_, n=14, BDI T_1_, n=15; SIAS T_1_, n=15.

^b^Significant at α=.01.

^c^Wilcoxon tests were carried out for the BLERT, GPTS-A, PANSS Positive, and TMT-B.

## Discussion

### Principal Findings

The main goal of this study was to evaluate the feasibility and acceptability of DiSCoVR, and to identify aspects in need of improvement [22]. We found that participants and therapists were generally satisfied with the intervention. The interactive role-play exercises were most commonly named as a strength of the intervention, as well as the opportunity to practice with social situations and the combination of VR and a therapist. Both therapists and participants provided useful feedback for further development, particularly regarding technical issues. A secondary goal was to obtain an estimate of treatment effect sizes on various outcome domains. We found a significant improvement in emotion perception. However, no significant change was observed on the other measures, and most effect sizes were negligible to small.

As stated in the “Introduction” section, commonly used criteria to evaluate feasibility and acceptance are acceptability, implementation potential, practicality, and limited-efficacy testing [22]. Additional areas of interest regarding the feasibility of technological interventions are provided by the Technology Acceptance Model, which emphasizes perceived ease of use, perceived usefulness, and user attitudes toward the technology [23]. Finally, the Systems Usability Scale [24] was likewise developed to evaluate technological innovations, and enquire about effectiveness, efficiency, and user satisfaction. In the following, we will focus on these criteria to evaluate the feasibility and acceptability of DiSCoVR.

### Acceptability, User Attitudes, and Satisfaction

Participants gave positive ratings to the enjoyability of the intervention, its usefulness for daily social interactions, the combination of VR and a therapist, and the appropriateness of the difficulty level. The most important strength of the intervention, as indicated by both participants and therapists, was the opportunity to practice with interactive social situations resembling daily life. As such, we succeeded in our goal of creating a method to facilitate practice in realistic social situations. However, the use of new technology to accomplish this also has an important disadvantage, in the form of technical issues (particularly problems with sound settings) and limited capability of the software (eg, lack of sophisticated animations and group role-play features). Technical capability and reliability were the most important point of criticism from participants as well as therapists. While these technical limitations were troublesome, it is possible to address them in future iterations of the software.

Notably, some participants considered the emotion recognition module to be particularly useful, but an equal number considered it to be unnecessary. Given the considerable variation in baseline social cognitive ability, it is likely that some participants were relatively unimpaired in emotion perception. For them, the first module may have been unnecessary. Therefore, (VR) SCTs may need to take a modular approach, that is, emphasize different domains for different people, based on their needs.

### Demand and Perceived Usefulness

We found that we could recruit participants relatively quickly, and most of them completed the intervention. DiSCoVR met treatment needs for the majority of participants, but not for everyone, mainly because people did not learn (all) the things they had wanted to learn, or because their subjective progress fell short of expectations. From this, we can learn that DiSCoVR does meet a demand, but it remains important that therapists and participants communicate clearly and regularly on social goals and their feasibility.

### Implementation Potential, Practicality, and Perceived Ease of Use

The intervention could generally be delivered as intended, with therapists reporting protocol deviations (55/303, 18.2% of sessions) and technical issues (45/303, 14.9% of sessions) relatively rarely. Therapists also indicated that the software and treatment protocol were intuitive and easy to use, and praised the quality and availability of technical support. However, the average time taken to complete the intervention was approximately 50% longer than intended, possibly reflecting that twice-weekly sessions were impractical. Nonetheless, the majority of participants was satisfied with the intensity of DiSCoVR, and the frequency and number of sessions match similar VR studies [14,44] and previous SCT studies [45-47]. Moreover, research suggests that for cognitive training, higher treatment intensity may produce better outcomes [48].

### Limited-Efficacy Testing and (Perceived) Effectiveness

Common subjective effects of the intervention were enhanced social skills, improved emotion recognition, and increased confidence and assertiveness. While demonstrating efficacy was not the goal of this study, we observed a significant improvement in emotion perception (specifically in the Ekman 60 Faces Test), but we did not see a statistically significant or clinically relevant improvement in higher-order social cognitive domains. Thus, despite our reasoning that the use of VR would facilitate generalization to higher-order social processes, we observed neither a statistically significant nor a clinically relevant change in any of the measures of ToM.

Although this study was small and uncontrolled, our findings appear to be consistent with previous studies indicating that lower-order processes such as emotion perception can be improved more effectively than higher-order processes [5]. Perhaps this is because, more so than identification of emotions in static pictures, ToM requires synthesis of multiple processes and sources of information [49], such as identifying and remembering relevant contextual details and processing of emotional cues (eg, verbal, auditive, facial, body language).

Our second module may have involved too much emphasis on emotion perception and too little on higher-order reflective processes. While little is known about what it takes to improve ToM [50], a recent meta-analysis [8] suggested that SCTs encompassing multiple domains of social cognition may be more effective than targeted interventions. Therefore, going forward, a greater emphasis on integration of higher-order social cognitive processes and application in social situations may be necessary.

### Adjustments as a Result of the Pilot Study

To address concerns about the interaction module (Module 3), the graphical quality, character animations, and sound control settings of the software were updated. Given the lack of an effect on ToM, we have also updated the second VR module. We changed the multiple-choice questions into open-ended questions to incite in-depth reflection on avatars’ behavior, thoughts, and feelings. That is, instead of asking only how an avatar is feeling (from a 4-choice menu), we now also ask *why,* what they are thinking, and how this relates to their behavior. This way, we hope to stimulate integrative reflection on social situations and engagement of higher-order social cognition.

We also adjusted the treatment protocol, to stimulate practical application of social cognition in daily life, and to better align the intervention with participants’ treatment needs. First, we placed a stronger emphasis on the use and practice of strategies throughout the intervention: therapists were more explicitly instructed to select a strategy with participants before each VR session, and to encourage its application in homework exercises. Second, we simplified the way goals are set, reflected upon, and evaluated, allowing goals to not only exclusively target social cognition (eg, recognizing social cues better), but also social functioning (eg, making friends). This way, we aim to enhance the relevance and generalization of training content, to ensure treatment needs are met. Lastly, we have added a monthly supervision group where therapists can receive input and advice from the research team and one another.

### Limitations

As an uncontrolled pilot study, the results of this study lack statistical power and methodological rigor to draw conclusions concerning the efficacy of DiSCoVR. Moreover, parallel versions were unavailable (Ekman 60 Faces, BLERT, TMT, all questionnaires) or were not administered in a randomized order (TASIT, Faux Pas). We therefore cannot exclude the possibility of learning, repetition, and order effects. For example, administering TASIT-A before B has been shown to result in significantly higher scores on TASIT-A [31] than if B is administered before A. This could potentially obfuscate treatment effects, as in this study TASIT-A was administered first. In addition, our recruitment method required potential participants to have sufficient ability for reflection to recognize a need for social-cognitive treatment. Therefore, we may have failed to recruit people with more severe (social) cognitive deficits, limiting the generalizability of our findings. Finally, we did not use a standardized measure to assess feasibility and acceptability, such as the System Usability Scale [24] or the Simulator Sickness Questionnaire [51] because we were interested in elements specific to our intervention. Thus, while our custom survey was informative for further development, it cannot be directly compared with previous research.

### Conclusions

We set out to develop a new type of SCT, building upon existing interventions by using VR as a tool. Taking into account the criteria described above [22-24], we can conclude that VR SCT is feasible and acceptable for both patients and therapists, and captures the interactive nature of social situations. This pilot study therefore demonstrates that a larger-scale clinical trial using these research and treatment protocols is feasible and acceptable. However, this study also demonstrated that there is room for improvement, particularly regarding the content and reliability of the VR software and hardware. Based on these results, we have adjusted DiSCoVR. Our next step will be to test this adjusted version in a randomized controlled trial [52], comparing it with an active control condition (VR relaxation).

## Appendix

Multimedia Appendix 1 Order and length of measures.

## Abbreviations

BDI: Beck Depression Inventory
BLERT: Bell–Lysaker Emotion Recognition Test
DiSCoVR: Dynamic Interactive Social Cognition Training in Virtual Reality
EAT: Empathic Accuracy Task
GPTS: Green Paranoid Thought Scale
M.I.N.I. Plus: Mini International Neuropsychiatric Interview Plus
PANSS: Positive and Negative Syndrome Scale
RVP: Rapid Visual Processing
SCT: Social Cognition Training
SERS: Self-Esteem Rating Scale
SIAS: Social Interaction Anxiety Scale
TASIT: The Awareness of Social Inference Test
TMT: Trail Making Test
VR: virtual reality
