# Supplementary material for: Dynamic Interactive Social Cognition Training in Virtual Reality (DiSCoVR) for People With a Psychotic Disorder: Single-Group Feasibility and Acceptability Study
Source: JMIR Ment Health. 2020 Aug 7;7(8):e17808. doi: 10.2196/17808 (PMC7442939; doi:10.2196/17808)
Supplement: Multimedia Appendix 1 [file mental_v7i8e17808_app1.docx]

Appendix

Table A1: *Order and length of measures*.

|  |  | Length (minutes) | T0 | T1 | Parallel versions used |
| --- | --- | --- | --- | --- | --- |
| 1 | Demographic information | 3 | X |  |  |
| 2 | NLV (NART) | 2 | X |  |  |
| 3 | Evaluation of DiSCoVR | 15 |  | X |  |
| 4 | SIAS | 4 | X | X |  |
| 5 | GPTS | 4 | X | X |  |
| 6 | RVP | 7 | X | X |  |
| 7 | TMT | 4 | X | X |  |
| 8 | FEEST | 10 | X | X |  |
| 9 | BLERT | 10 | X | X |  |
| 10 | EAT | 15 | X | X | X |
| 11 | TASIT | 45 | X | X | X |
| 12 | Faux Pas | 20 | X | X | X |
| 13 | BDI | 4 | X | X |  |
| 14 | SERS | 4 | X | X |  |
| 15 | MINI | 20 | X |  |  |
| 16 | PANSS | 25 | X | X |  |
